# Remarkable spatial variation in the seroprevalence of *Coxiella burnetii* after a large Q fever epidemic

**DOI:** 10.1186/s12879-017-2813-y

**Published:** 2017-11-21

**Authors:** Roan Pijnacker, Johan Reimerink, Lidwien A. M. Smit, Arianne B. van Gageldonk-Lafeber, Jan-Paul Zock, Floor Borlée, Joris Yzermans, Dick J. J. Heederik, Catharina B. M. Maassen, Wim van der Hoek

**Affiliations:** 1 Centre for Infectious Disease Control, National Institute for Public Health and the Environment, (RIVM), PO Box 1, 3720 BA Bilthoven, the Netherlands; 2 European Programme for Intervention Epidemiology Training (EPIET), European Centre for Disease Prevention and Control, (ECDC), Stockholm, Sweden; 3 Institute for Risk Assessment Sciences, Utrecht University, Utrecht, the Netherlands; 4 Netherlands Institute for Health Services Research, (NIVEL), Utrecht, the Netherlands

**Keywords:** *Coxiella burnetii*, Q fever, Risk factors, Seroprevalence, Zoonosis

## Abstract

**Background:**

Prior to the 2007–2010 Q fever epidemic in the Netherlands, the seroprevalence of antibodies against *Coxiella burnetii* in the general population was 1.5%, which is low compared to other countries. We aimed to determine the seroprevalence after the Q fever epidemic among people living in the affected area, compare the seroprevalence with the incidence of Q fever notifications during the 2007–2010 Q fever epidemic, and to identify farm exposures associated with having antibodies against *C. burnetii*.

**Methods:**

During the period March 2014–February 2015, residents aged 18–70 years from two provinces were invited by general practitioners to complete a questionnaire on their symptoms and personal characteristics and to submit a blood sample. We used the mandatory provincial database of livestock licences to calculate distance to farms/farm animals for each participant. To compare ELISA-positive participants for *C. burnetii* antibodies with those who were negative, we calculated prevalence ratios (PR) using binominal regression. We compared the *C. burnetii* seroprevalence in the period March 2014–February 2015 with the incidence of Q fever notifications during the 2007–2010 Q fever epidemic at municipal level by calculating the Spearman correlation coefficient.

**Results:**

Of the 2296 participants (response rate: 34%), 6.1% (*n* = 139, 95% CI 5.1–7.1%) had *C. burnetii* antibodies (range in municipalities: 1.7–14.1%). *C. burnetii* seroprevalence was higher in individuals living within 1000 m of goat farms (PR 3.0; 95% CI 1.4–6.4) or within 1000 m of > 50 goats (PR 1.9; 95% CI 1.2–3.0). Seroprevalence increased with decreasing distance to the closest goat farm that was infected during the epidemic years (< 500 m, PR 9.5, 95% CI 2.8–32; 500–1000 m, PR 4.5, 95% CI 2.6–7.7; 1000–1500 m, PR 2.2, 95% CI 1.1–4.3, 1500–2000 m, PR 1.2, 95% CI 0.6–2.5; > 2000 reference group). There was no significant correlation between *C. burnetii* seroprevalence and Q fever incidence during the 2007–2010 epidemic (*r*_*s*_ = 0.42, *p* = 0.156).

**Conclusions:**

Results showed a remarkable spatial variation in *C. burnetii* seroprevalence in a relatively small livestock dense area. It confirms previous evidence that the Q fever epidemic was primarily the result of airborne *C. burnetii* transmission from Q fever affected goat farms.

## Background

Q fever is a zoonosis caused by the intracellular bacterium *Coxiella burnetii*. The most common reservoirs for *C. burnetii* are goats, sheep and cattle, although a variety of other species can get infected [1]. In goats and sheep, the main clinical symptom of Q fever is abortion and in cattle reduced fertility but most animals remain asymptomatic. Animals shed *C. burnetii* in milk, faeces, urine and especially in birth materials [2]. Humans typically acquire the infection through the inhalation of contaminated aerosols, with approximately 60% of the infected remaining asymptomatic [3, 4]. In symptomatic patients, acute Q fever usually presents as an influenza-like febrile illness, pneumonia, or hepatitis.

The 2007–2010 Q fever epidemic in the Netherlands with over 4000 notified human cases was a major public health event [5] and resulted in increased concern about possible health risks for the general population living in livestock dense areas [6]. The epicentre of the Q fever epidemic was in the province of Noord-Brabant, which in 2007 had particularly high densities of poultry (5024 animals/km^2^), cattle (125 animals/km^2^), goats (23 animals/km^2^) and sheep (20 animals/km^2^) [7]. Since 2009, there is an ongoing mandatory annual vaccination programme for dairy goats and dairy sheep on farms with more than 50 animals and the number of acute Q fever notifications is back at the level it was before 2007 [8].

Prior to the Q fever epidemic, the seroprevalence was estimated at 1.5% in the general population of the Netherlands in 2006 using an enzyme-linked immunosorbent assay (ELISA) [9]. This was corrected to 2.4% by confirmation on a subset using immunofluorescence assay (IFA), which is considered the reference method for diagnostic screening for *C. burnetii* antibodies. Even 2.4% is a low seroprevalence figure compared to many other countries. For example, a community-based study conducted in the USA showed a seroprevalence of 3.1% using IFA [10]. A study among blood donors in France in 1988 and in Japan in the late 1990s showed a seroprevalence of 4.0% and 3.6%, respectively, both using IFA [11, 12]. The dynamics of antibodies against *C. burnetii* and the role of changing or repeated exposure, are still poorly understood. Historically, there is evidence that the seroprevalence was much higher in the Netherlands in the 1980s [17].

To gain more insight in the dynamics of *C. burnetii* seroprevalence, we conducted a serological survey for antibodies against *C. burnetii* among people living in a livestock-dense area in the south of the Netherlands where the epidemic occurred. The aims were to 1) determine the seroprevalence of antibodies against *C. burnetii* among people living in the area affected by the Q fever epidemic several years after the epidemic; 2) compare the *C. burnetii* seroprevalence with the incidence of Q fever notifications during the 2007–2010 Q fever epidemic; and 3) to identify farm exposures associated with having antibodies against *C. burnetii*.

## Methods

### Study design and population

This cross-sectional population-based serological survey took place as part of the ‘Livestock Farming and Neighbouring Residents’ Health’ study (VGO). For details about the recruitment of the VGO population, we refer to previously published papers [13, 14]. Briefly, a survey was conducted among 14,163 adults aged 20–72 years from the general population after a two-stage selection procedure. First, general practitioners were recruited and selected based on registration quality criteria; then all patients of the selected general practitioners were invited [13]. Participants who gave their consent for further contact for additional studies and who were not working or living on a farm, were eligible for the medical survey. Based on their home addresses, 12 temporary research centres were established. All participants living within approximately 10 km of a temporary research centre were invited to participate in the study (a total of 7180 people aged 20–72 years) [14]. Of those, 2494 (response rate 35%) participated in a medical examination at one of 12 temporary study centres. Serum samples were obtained from 2422 participants.

### Animal exposure assessment

Farm locations and number and type(s) of animals per farm were obtained from the provincial database of mandatory environmental licences for keeping livestock (BVB) for the year 2012. These data were used to calculate the distances to farms for each participant based on their residential address. Several exposure variables were defined, such as the presence of animal farms (binary), and the total number of sheep, goat (categorized as more or less than 50 goats/sheep, i.e. the cut-off for mandatory vaccination) and cattle (tertiles) within 500 m and 1000 m of the participants’ residential address. Exposure was based on the presence of a Q fever affected dairy goat farm near the residential address (categorized as < 500 m, 500–1000 m, 1000–1500 m, 1500–2000 m, with > 2000 m being the reference). A dairy goat farm was considered affected when it had experienced abortion waves between 2005 and 2009 (data provided by the GD Animal Health) or when they tested positive for Q fever in the mandatory bulk tank milk monitoring system using PCR for the detection of *C. burnetii* DNA that was implemented in 2009 and is still ongoing (data from the Food and Consumer Product Safety Authority). Q fever on farms experiencing abortion waves was confirmed with immunohistochemistry.

### Data collection

**Fig. 1:**
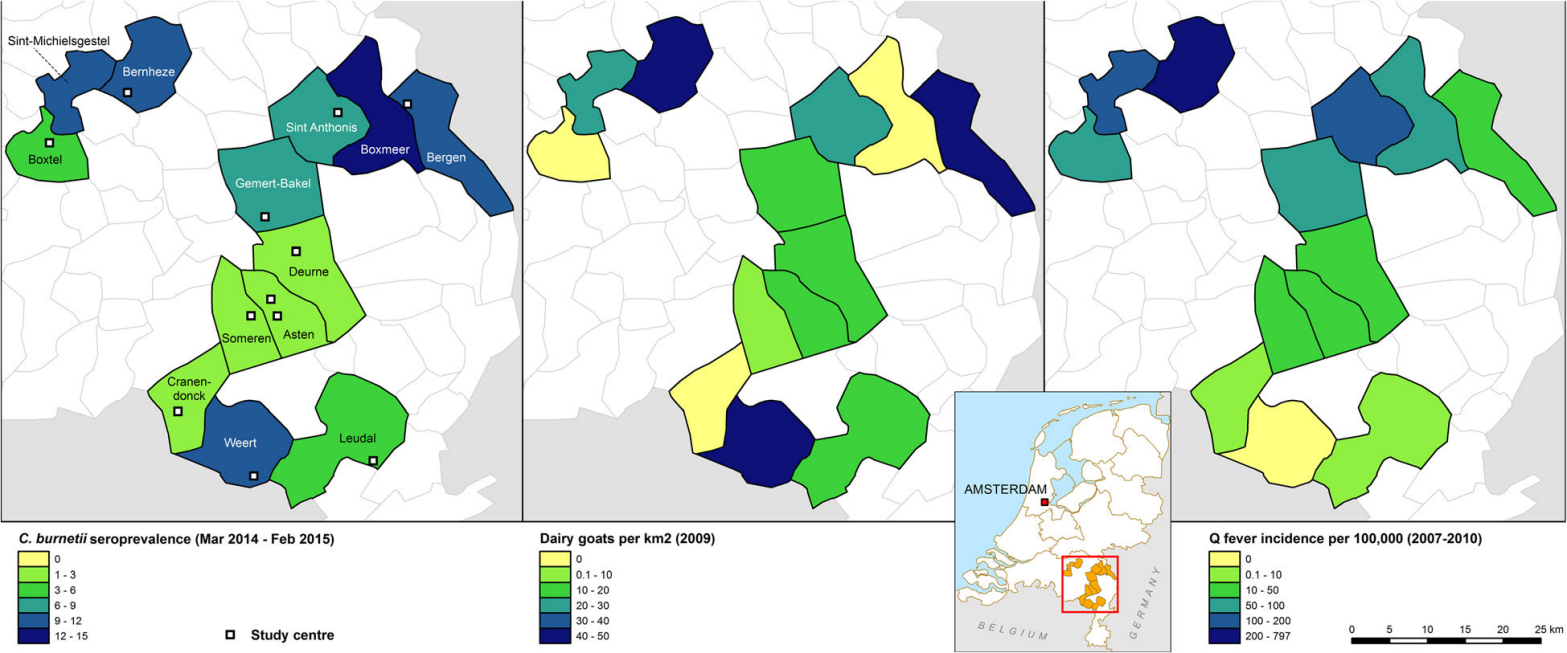
The seroprevalence of antibodies against *C. burnetii* in March 2014 to February 2015, dairy goat density per km^2^ in 2009, and the incidence of Q fever notifications in the period 2007–2010, by municipality, region Noord-Brabant and the northern part of region Limburg, the Netherlands

Subjects completed a questionnaire including items on respiratory health, residential characteristics, smoking habits, education, occupation, and animal contact. Questionnaire data and serum samples were collected from 10 March 2014 to 27 February 2015 [15]. All study centres were in the eastern part of the province of Noord-Brabant and the northern part of the province of Limburg (Fig. 1). These were the provinces most affected by the Q fever epidemic [5].

### Q fever notifications

Q fever is a notifiable disease in the Netherlands and information on human data is stored in an electronic database at the National Institute for Public Health and the Environment (RIVM). We used data on human Q fever cases that were notified during the 2007–2010 Q fever epidemic. Cases were assigned to a municipality based on the participants’ place of residence.

### Laboratory analysis

Sera were analysed for the presence of IgG antibodies to *C. burnetii* phase II antigen, using a commercial ELISA (Serion ELISA classic, Virion/Serion, Würzburg, Germany). IgG antibodies were measured quantitatively and the results generated from standard curves were reported in International Units/ml. In line with manufacturer’s recommendations, samples with values of < 20 IU/ml were considered negative, values of 20–30 IU/ml were scored as borderline, and those that had values of > 30 IU/ml were considered as positive.

### Statistical analysis

Based on the participants’ place of residence, we calculated the seroprevalence of antibodies against *C. burnetii* by municipality. Municipalities with less than 10 participants were excluded. The *chi*-square test was used to test for differences in *C. burnetii* seroprevalence between municipalities. The relationship between the seroprevalence of antibodies against *C. burnetii* and the incidence of Q fever notifications during the 2007–2010 Q fever epidemic was assessed at municipal level by calculating the Spearman correlation coefficient. A *p*-value of < 0.05 was considered significant. We examined the association between farm exposures and *C. burnetii* seroprevalence using log-binominal regression models and calculated adjusted prevalence ratios (PRs) for the outcome ELISA positive and borderline combined versus ELISA negative. We incorporated cluster-robust standard errors to account for clustering at study centre level. Analyses were adjusted for age (categorized as < 40, 40–60 and > 60 years old), gender, educational level (categorized as low, middle and high) and smoking (ever smoked, yes/no). The presence of a certain type of animal farm was adjusted for the presence of farms with other animal species. The number of farm animals of a species was adjusted for the presence of other farm animal species. Co-linearity between independent variables was checked prior to regression analysis. Participants who had moved to the study area after 2010 were excluded. In a sensitivity analysis, samples with borderline values of 20–30 IU/ml were excluded. Statistical analyses were performed with STATA v13.0 software (StataCorp, College Station, Texas).

## Results

The overall seroprevalence of IgG antibodies against phase II of *C. burnetii* was 6.1% (*n* = 139, 95% CI 5.1–7.1). The median age for participants positive for antibodies against *C. burnetii* was 59 years (interquartile range [IQR] 48–66 years old); 76 (52.1%) of them were male. A total of 126 participants were excluded because they had moved to the study area after 2010.

### Spatial distribution

The spatial distribution of goats, incidence of Q fever notifications and seroprevalence of antibodies against *C. burnetii* are displayed in Figure 1. At municipality level, the seroprevalence of antibodies against *C. burnetii* ranged from 1.7 to 14.1% and was significantly different between municipalities (*p* < 0.001) (Table 1). There was a positive correlation between *C. burnetii* seroprevalence and Q fever incidence during the 2007–2010 epidemic but this was not significant (*r*_*s*_ = 0.42, *p* = 0.156).

**Table 1:** Q fever incidence in the period 2007–2010 and serological results for antibodies against *C. burnetii*, by municipality, the Netherlands, 10 March 2014–27 February 2015^a^

Municipality^b^	Population	Q fever incidence in the period 2007–2010^c^	*C. burnetii* positive/sera tested	*C. burnetii* seroprevalence (95% CI^d^)
Boxmeer	28,610	87	10/71	14.1 (7.0–24.4)
Weert	48,330	0	18/164	11.0 (6.6–16.8)
Bernheze	29,620	797	32/301	10.6 (7.3–14.7)
Bergen	13,400	37	4/38	10.5 (2.9–24.8)
Sint-Michielsgestel	28,270	195	4/40	10.0 (2.8–23.7)
Sint Anthonis	11,790	136	22/288	7.6 (4.8–11.3)
Gemert-Bakel	28,510	95	21/308	6.8 (4.3–10.2)
Boxtel	30,280	73	6/149	4.0 (1.5–8.6)
Leudal	36,750	3	5/134	3.7 (1.2–8.5)
Someren	18,230	16	5/175	2.9 (0.9–6.5)
Deurne	31,470	13	2/104	1.9 (0.2–6.8)
Asten	16,360	37	6/326	1.8 (0.7–4.0)
Cranendonck	20,270	5	3/174	1.7 (0.4–5.0)
Overall	527,600	123	139/2296	6.1 (5.1–7.1)

^a^Spearman correlation coefficient between Q fever incidence during the 2007–2010 epidemic and *C. burnetii* seroprevalence was not significant (*rs* = 0.42, *p* = 0.156)

^b^Municipalities with < 10 participants are not displayed

^c^Per 100,000 population

^d^95% confidence interval (CI)

### Risk factors for *C. burnetii* infection

Seroprevalence of IgG antibodies against phase II of *C. burnetii* was higher among those residing within 1000 m of a goat farm (prevalence 15%, PR 3.0; 95% CI 1.4–6.4) or within 1000 m of more than 50 goats (prevalence 11%, PR 1.9; 95% CI 1.2–3.0) (Table 2). Residing within 1000 m of a goat farm was still associated with seroprevalence when excluding Q fever affected goat farms (data not shown). Seroprevalence increased with decreasing distance from the residential address to the closest Q fever affected goat farm (< 500 m, prevalence 50%, PR 9.5, 95% CI 2.8–32; 500–1000 m, prevalence 25%, PR 4.5, 95% CI 2.6–7.7; 1000–1500 m, prevalence 12%, PR 2.2, 95%CI 1.1–4.3, 1500–2000 m, prevalence 7.0%, PR 1.2, 95% CI 0.6–2.5; > 2000 reference group, prevalence 5.4%). The strength of the association did not change in stratified analysis by goat farms that experienced abortion storms and goat farms that only tested positive for *C. burnetii* in bulk milk samples (data not shown). Exposure variables related to sheep and cattle were not significantly associated with the presence of antibodies against *C. burnetii*.

**Table 2:** Adjusted prevalence ratios and corresponding 95% confidence intervals of the association between the presence of antibodies against *C. burnetii*, and characteristics/exposures, the Netherlands, March 2014–February 2015

	Participants (%) (*n* = 2296)	Prevalence ratio (95% CI)^a^	*p*-value
Age			
< 40 years old	177 (7.7)	Reference	
40–60 years old	1045 (45.5)	1.0 (0.5–1.8)	0.93
> 60 years old	1074 (46.8)	1.1 (0.7–1.7)	0.77
Ever smoked	1262 (55.1)	0.8 (0.6–1.0)	0.09
Female gender	1240 (54.0)	0.8 (0.6–0.9)	**0.01**
Educational level			
Low	592 (25.8)	Reference	
Middle	1027 (44.7)	0.8 (0.6–1.0)	**0.03**
High	677 (29.5)	0.7 (0.4–1.0)	0.06
Animal farms			
Presence of Q fever affected goat farm^b^			
within > 2000 m	2038 (88.8)	Reference	
within 1500–2000 m	139 (6.1)	1.2 (0.6–2.5)	0.53
within 1000–1500 m	91 (4.0)	2.2 (1.1–4.3)	**0.02**
within 500–1000 m	24 (1.1)	4.5 (2.6–7.7)	**< 0.01**
within < 500 m	4 (0.2)	9.5 (2.8–31.6)	**< 0.01**
Presence of goat farm (yes/no)			
within 1000 m^b^	159 (6.9)	3.0 (1.4–6.4)	**< 0.01**
within 500 m^c^	40 (1.7)	2.0 (0.7–5.7)	0.19
Presence of sheep farm (yes/no)			
within 1000 m^b^	391 (17.0)	1.4 (0.7–2.8)	0.28
within 500 m^c^	120 (5.2)	1.2 (0.5–3.2)	0.69
Presence of cattle farm (yes/no)			
within 1000 m^b^	2154 (93.8)	1.2 (0.5–3.3)	0.69
within 500 m^c^	1130 (49.2)	1.1 (0.9–1.4)	0.18
Farm animals^d^			
> 50 goats within 1000 m	249 (10.8)	1.9 (1.2–3.0)	**< 0.01**
> 50 sheep within 1000 m	619 (27.0)	1.2 (0.7–2.0)	0.43
Number of cattle within 1000 m (tertiles)			
≤ 314	752 (32.8)	Reference	
315–834	766 (33.4)	1.0 (0.6–1.6)	1.00
≥ 835	778 (33.9)	1.0 (0.7–1.5)	0.85

^a^Adjusted for gender, age (categorised as < 40, 40–60 and > 60 years old), educational level (low, middle and high), and smoking (ever smoked? yes/no)

^b^Adjusted for the presence of other animal farms within 1000 m (goat, sheep and cattle)

^c^Adjusted for the presence of other animal farms within 500 m (goat, sheep and cattle)

^d^Adjusted for the presence of other farm animals (goat, sheep and cattle)

entries in bold are all *p*-values

### Sensitivity analysis

In sensitivity analysis, 48 participants with ELISA borderline results were excluded. A decreasing distance to the closest Q fever affected goat farm was still associated with increasing seroprevalence (<500 m, prevalence 50%, PR 13.7, 95% CI 4.8–39; 500–1000 m, prevalence 14%, PR 3.7, 95% CI 1.9–7.3; 1000–1500 m, prevalence 7.0%, PR 1.9, 95% CI 1.2–3.2; 1500–2000 m, prevalence 3.0%, PR 0.8, 95% CI 0.4–1.7; >2000 m reference group, prevalence 3.8%).

However, the presence of goat farms or more than 50 goats within 1000 m of the participants’ residential address were no longer significantly associated with an increased prevalence of antibodies against *C. burnetii* (prevalence 7.5%, PR 2.0; 95% CI 0.7–5.5 and prevalence 5.5%, PR 1.4; 95% CI 0.8–2.3, respectively). Variables related to exposure to sheep and cattle were not significantly associated with antibodies against *C. burnetii*.

## Discussion

We found significant differences in the seroprevalence of antibodies against *C. burnetii* among municipalities in a relatively small livestock-dense area, ranging from 1.7 to 14.1% between municipalities. The seroprevalence of antibodies against *C. burnetii* was significantly associated with living close to goats and goat farms, especially those farms affected by Q fever during the 2007–2010 Q fever epidemic.

Residential proximity to goats thus provides an explanation for the spatial variation. However, the seroprevalence of *C. burnetii* antibodies in some of the municipalities was lower than expected in this livestock-dense area. Some of the municipalities had a seroprevalence that was equal or lower than the seroprevalence in the general population before the Q fever epidemic from 2007 to 2010 [9]. In 2006, 1.5% of the general population tested positive for *C. burnetii* antibodies using the same diagnostic test as the current study. The seroprevalence in the current study is probably an under-estimation of the actual number of infections that occurred during the outbreak. First, the ELISA used in the current study has a lower sensitivity and similar specificity compared with the IFA, which is considered the reference method for diagnostic screening of *C. burnetii* antibodies [16]. For instance, the ELISA seroprevalence estimate of 1.5% in the general population in 2006 in the Netherlands was adjusted to 2.4%, based on confirmation with IFA results in a sub-set [9]. Second, Q fever patients can sero-revert from IFA-positive to IFA-negative in the years after a Q fever outbreak [17, 18]. In a study conducted in 2014 in the general adult population in the village where the first Q fever outbreak was reported, with a seroprevalence of 33.8%, 16.9% of the participants sero-reverted from IFA-positive to IFA-negative in the years after the outbreak [17]. The temporal dynamics of *C. burnetii* seroprevalence is difficult to interpret, as demonstrated by a study of secular trends in *C. burnetii* antibody prevalence in the Netherlands, which showed much higher seroprevalence in the general population in the 1980s [19].

Earlier studies already identified that living close to goat farms is the most important risk factor for Q fever [20–22]. In the present study, these findings were confirmed. Moreover, the association with seroprevalence was even stronger for Q fever affected goat farms. This is plausible, as a high number of *C. burnetii* are shed with abortion, resulting in increased human exposure [2]. This was underlined by the increasing seroprevalence with decreasing distance from the residential address to the closest Q fever affected goat farm. We expected a stronger association with the presence of *C. burnetii* antibodies for goat farms that experienced abortions than for farms that were only positive in tank milk monitoring. However, some farms that tested positive in tank milk had also experienced abortion waves. Furthermore, the last farm with Q fever-induced abortions was in 2009 while the last goat farm was declared negative only in 2016. The association between living close to goat farms or with more than 50 goats within 1000 m from the residential address and having *C. burnetii* antibodies was no longer significant in sensitivity analysis although it showed a positive association. This is probably due to decreased statistical power as participants with borderline ELISA results were excluded. Moreover, the increasing seroprevalence with decreasing distance from the residential address to the closest Q fever affected goat farm was significant in the sensitivity analysis.

Exposure variables related to cattle were not associated with the seroprevalence of antibodies against *C. burnetii*. This could be explained by differences in *C. burnetii* shedding patterns that may account for the more frequent identification of goats than cattle as source of human Q fever [23]. While the main clinical symptom of Q fever in cattle is infertility, Q fever in goats is mainly associated with abortion. As previously mentioned, birth products are associated with larger numbers of *C. burnetii*, while smaller numbers are excreted in urine, faeces and milk [2]. Human exposure to *C. burnetii* from cattle might therefore be limited, although more than half (57%) of 344 dairy cattle herds tested positive for *C. burnetii* using PCR on bulk tank milk in the Netherlands in the period 2005–2006 [20]. In line with our findings, isolates from cattle showed different genotypes than those in goats, sheep and humans in the Netherlands during the Q fever epidemic, although data is sparse [24].

No association was found between sheep-related exposure variables and the presence of antibodies against *C. burnetii*. This is probably due to the lower number of sheep farms that were bulk milk-positive for *C. burnetii* using PCR compared with goat farms. The implemented mandatory bulk milk monitoring scheme that was carried out since October 2009 indicated that 96 (27%) large dairy goat farms and 2 (5%) dairy sheep farms had tested bulk milk-positive for *C. burnetii* by April 2011 [25, 26].

Although the *C. burnetii* seroprevalence was positively correlated with Q fever incidence during the period 2007–2010 at municipality level, this correlation was not significant. This was mainly due to one municipality, Weert, where the seroprevalence was high, while no human Q fever cases were reported from that municipality during the epidemic. When we excluded this municipality, the Spearman correlation between the *C. burnetii* seroprevalence and Q fever incidence during the period 2007–2010 was significant (*r*_*s*_ = 0.75, *p* = 0.005). Excluding any of the other municipalities did not alter the Spearman correlation coefficient. There were Q fever affected dairy goat farms in and around the municipality of Weert. The possible reasons for the lack of Q fever cases while the observed *C. burnetii* seroprevalence was high included: 1) lower infectious doses emitted from the farms; 2) local environmental conditions that were less conducive for transport of bacteria through the air at the time of the abortion storm on the farm; 3) a less virulent strain of *C. burnetii* than elsewhere; and 4) under-diagnosis and/or under-reporting of human cases by general practitioners. An earlier study reported that each acute Q fever notification represented ≥ 12 incident Q fever infections [27]. This is likely primarily due to asymptomatic infections, undiagnosed symptomatic infections and laboratory-confirmed infections that did not fulfil the clinical criteria for notification.

## Conclusions

The study indicates remarkable spatial variation in *C. burnetii* seroprevalence within a relatively small area. This variation can largely be attributed to differences in transmission intensity during the 2007–2010 epidemic. Our results add to the pool of evidence that the Q fever epidemic was primarily the result of airborne *C. burnetii* transmission from Q fever affected goat farms. Although the vaccination of goats has shown to be effective in reducing shedding of bacteria, *C. burnetii* can survive in the environment for years and has been detected in ambient air after the Q fever outbreak [28]. Therefore, physicians should remain vigilant for human Q fever cases despite the marked decrease in notifications since the epidemic.

## Abbreviations

CI: Confidence interval
ELISA: enzyme-linked immunosorbent assay
IFA: Immunofluorescence assay
IQR: Interquartile range
PR: Prevalence ratio
RIVM: National Institute for Public Health and the Environment (Dutch acronym)
VGO: Livestock Farming and Neighbouring Residents’ Health (VGO) study (Dutch acronym)
